# Development of a novel method for measuring tissue oxygen pressure to improve the hypoxic condition in subcutaneous islet transplantation

**DOI:** 10.1038/s41598-022-19189-2

**Published:** 2022-08-30

**Authors:** Hiroaki Mitsugashira, Takehiro Imura, Akiko Inagaki, Yukiko Endo, Takumi Katano, Ryusuke Saito, Shigehito Miyagi, Kimiko Watanabe, Takashi Kamei, Michiaki Unno, Masafumi Goto

**Affiliations:** 1 Department of Surgery, Tohoku University Graduate School of Medicine, Sendai, 980-0872 Japan; 2 Division of Transplantation and Regenerative Medicine, Tohoku University Graduate School of Medicine, 2-1 Seiryo-machi, Aoba-ku, Sendai, Miyagi 980-8575 Japan

**Keywords:** Medical research, Type 1 diabetes

## Abstract

Subcutaneous tissue is a promising site for islet transplantation, but poor engraftment, due to hypoxia and low vascularity, hinders its prevalence. However, oxygen partial pressure (pO_2_) of the subcutaneous space (SC) and other sites were reported to be equivalent in several previous reports. This contradiction may be based on accidental puncture to the indwelling micro-vessels in target tissues. We therefore developed a novel optical sensor system, instead of a conventional Clark-type needle probe, for measuring tissue pO_2_ and found that pO_2_ of the SC was extremely low in comparison to other sites. To verify the utility of this method, we transplanted syngeneic rat islets subcutaneously into diabetic recipients under several oxygenation conditions using an oxygen delivery device, then performed pO_2_ measurement, glucose tolerance, and immunohistochemistry. The optical sensor system was validated by correlating the pO_2_ values with the transplanted islet function. Interestingly, this novel technique revealed that islet viability estimated by ATP/DNA assay reduced to less than 75% by hypoxic condition at the SC, indicating that islet engraftment may substantially improve if the pO_2_ levels reach those of the renal subcapsular space. Further refinements for a hypoxic condition using the present technique may contribute to improving the efficiency of subcutaneous islet transplantation.

## Introduction

Type 1 diabetes (T1D) is a chronic autoimmune disease caused by the destruction of insulin-producing pancreatic beta-cells^1^. Insulin therapy cannot completely normalize glucose homeostasis despite significant technological improvements. Therefore, curative therapy for T1D has relied on the replacement of beta-cells by transplantation. Whole organ pancreas transplantation is a powerful therapeutic option for T1D, but has received limited acceptance because of substantial surgical invasiveness, perioperative complications, and—in some countries—fewer opportunities^2–4^. In contrast, islet transplantation represents a clinically viable option to achieve long-term insulin independence for T1D patients with advances in islet isolation^5–7^ and posttransplant management^8–10^. Compared to whole organ transplantation, it offers a promising minimally invasive approach without surgical complications and has already been established as a treatment option for severe T1D^3,8,11^. At present, intraportal islet injection is regarded as an established procedure for clinical islet transplantation. However, this method has several drawbacks, including the risk of portal vein embolism^12,13^, the occurrence of strong innate immune reactions^14–18^, and difficulty in removing transplanted islet grafts when needed in the case of stem cell-derived beta cell replacement.

Thus, more ideal transplant sites are strongly desired in the field of islet transplantation. The subcutaneous space (SC) is one such candidate and has several advantages, including minimal invasiveness, enough space for a large number of cells, easy site monitoring^19,20^, and early detection and removal of malignant growth if the medical need arises. However, despite these advantages, poor engraftment hinders its prevalence because of hypoxia and low vascularity relative to the conventional intrahepatic site^21^. Islets were encountered with oxidative stress from isolation^22,23^, and after subcutaneous transplantation, islets are quite sensitive to hypoxia and require an adequate oxygen supply until the graft is revascularized^24,25^. Several methods have been introduced to supply oxygen to islets transplanted at the SC, to maintain transplanted cell viability and improve glucose tolerance^26–29^.

In previous reports, despite the differences between rats and primates, the oxygen partial pressure (pO_2_) at the SC was not significantly different from that at other transplant sites (e.g., intravenous or renal subcapsular space [RS])^28,30,31^. A needle-type probe has thus far been used to measure pO_2_ by puncturing and inserting it into the SC; however, incorrect measurements may occur due to bleeding by puncture, atmospheric contamination from the surroundings, and pO_2_ measurement of micro-vessels. Considering the low vascularity, the former pO_2_ values at the SC are questionable, and in terms of oxygen concentration, the poor engraftment of subcutaneous islet transplantation cannot be fully explained. One possible explanation for this discrepancy is that the pO_2_ values at the SC are overestimated in most cases when a needle-type probe was used, most likely due to accidental puncture of the subcutaneous micro-vessels.

Therefore, in the present study, we aimed to develop a novel method for accurately measuring tissue oxygen pressure to solve the problems of previous methods with the puncture procedure. To achieve our purpose, we focused on the optical sensor system, instead of a conventional Clark-type needle probe, which is the current gold standard for in vivo oxygen measurement. The optical sensor system can theoretically make it possible to realize a non-contact method with target tissues. We verified the utility of this novel technique by measuring the pO_2_ values at the SC and by evaluating the islet graft function and/or viability after subcutaneous islet transplantation under several oxygenation conditions using an implantable oxygen delivery device.

## Results

### Comparison of pO_2_ between the subcutaneous space and renal subcapsular space by the non-contact method vs the direct contact method

**Figure 1:**
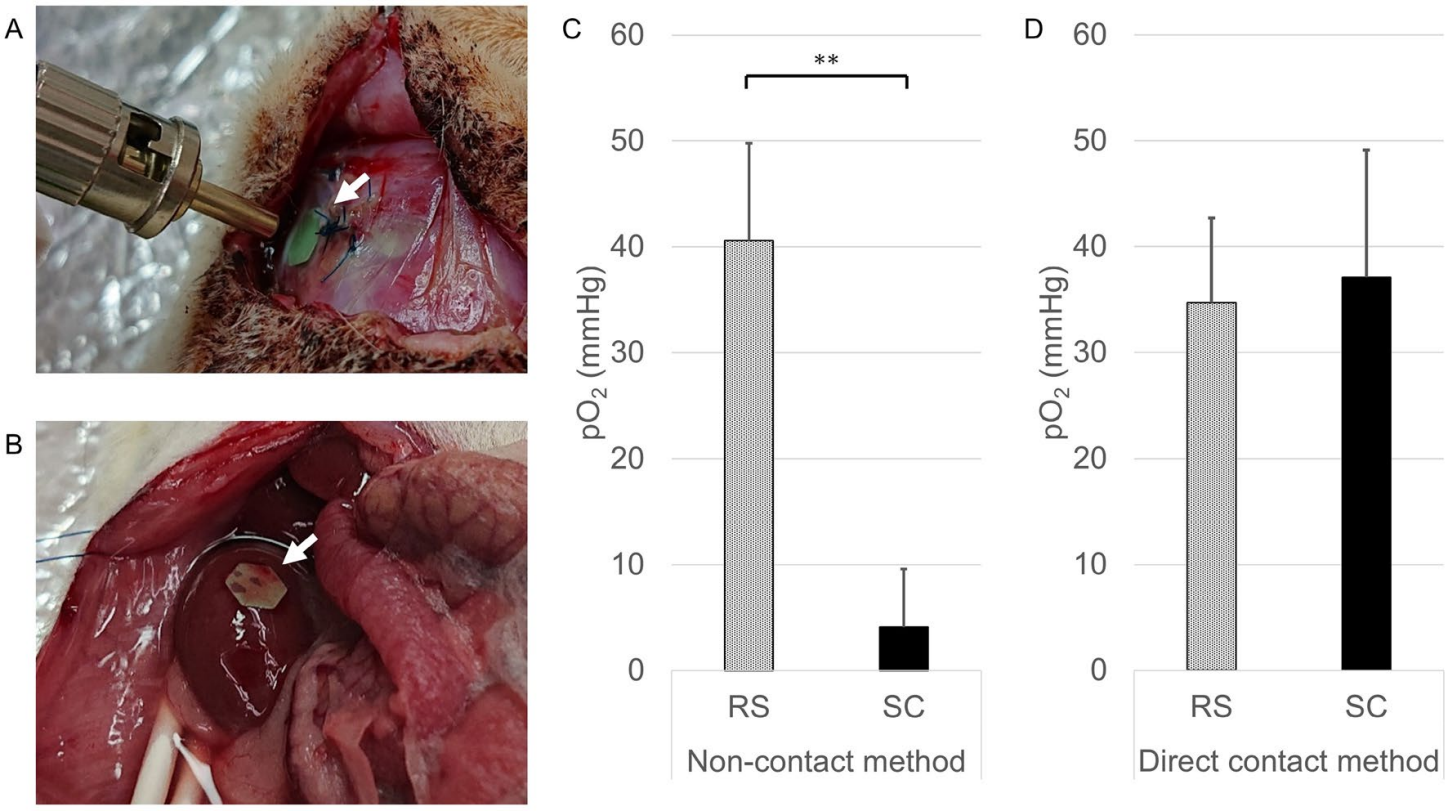
Comparison of pO_2_ between the subcutaneous space (SC) and renal subcapsular space (RS) using the optical sensor system. (**A**) Photograph of pO_2_ measurement at the SC using a non-contact-type optical oxygen sensor implanted under the subcutaneous capsule (arrows). (**B**) Photograph of pO_2_ measurement at the RS using a non-contact-type optical oxygen sensor implanted under the renal capsule (arrows). (**C**) Comparison of pO_2_ between SC and RS using a non-contact-type optical oxygen sensor. The pO_2_ of the SC group (black bar; 4.1 ± 5.5 mmHg, n = 10) was significantly lower than that of the RS group (dotted bar; 40.6 ± 9.2 mmHg, n = 5). **p < 0.01 (Student's *t* test). (**D**) Comparison of pO_2_ between the SC and RS using a direct contact-type optical oxygen sensor. No difference was observed between the SC (black bar; 37.1 ± 12.0 mmHg, n = 11) and RS (dotted bar; 34.7 ± 8.0 mmHg, n = 9). p = 0.62 (Student's *t* test).

Approximately 1 week after implantation of the sensor, no bleeding or inflammation was observed around the biological capsule surrounding the sensor at either the SC or RS (Fig. 1A,B). In the present study, pO_2_ was measured using an optical sensor system with or without contact with the target tissues. In the non-contact method, the pO_2_ of the SC group was significantly lower than that of the RS group (Fig. 1C, 4.1 ± 5.5 mmHg (n = 10) vs. 40.6 ± 9.2 mmHg (n = 5); p < 0.01). In contrast, no difference was observed in the direct contact method (Fig. 1D, 37.1 ± 12.0 mmHg (n = 11) vs. 34.7 ± 8.0 mmHg (n = 9); p = 0.62).

### Influence of the interjacent subcutaneous capsule on the optical sensor system

**Figure 2:**
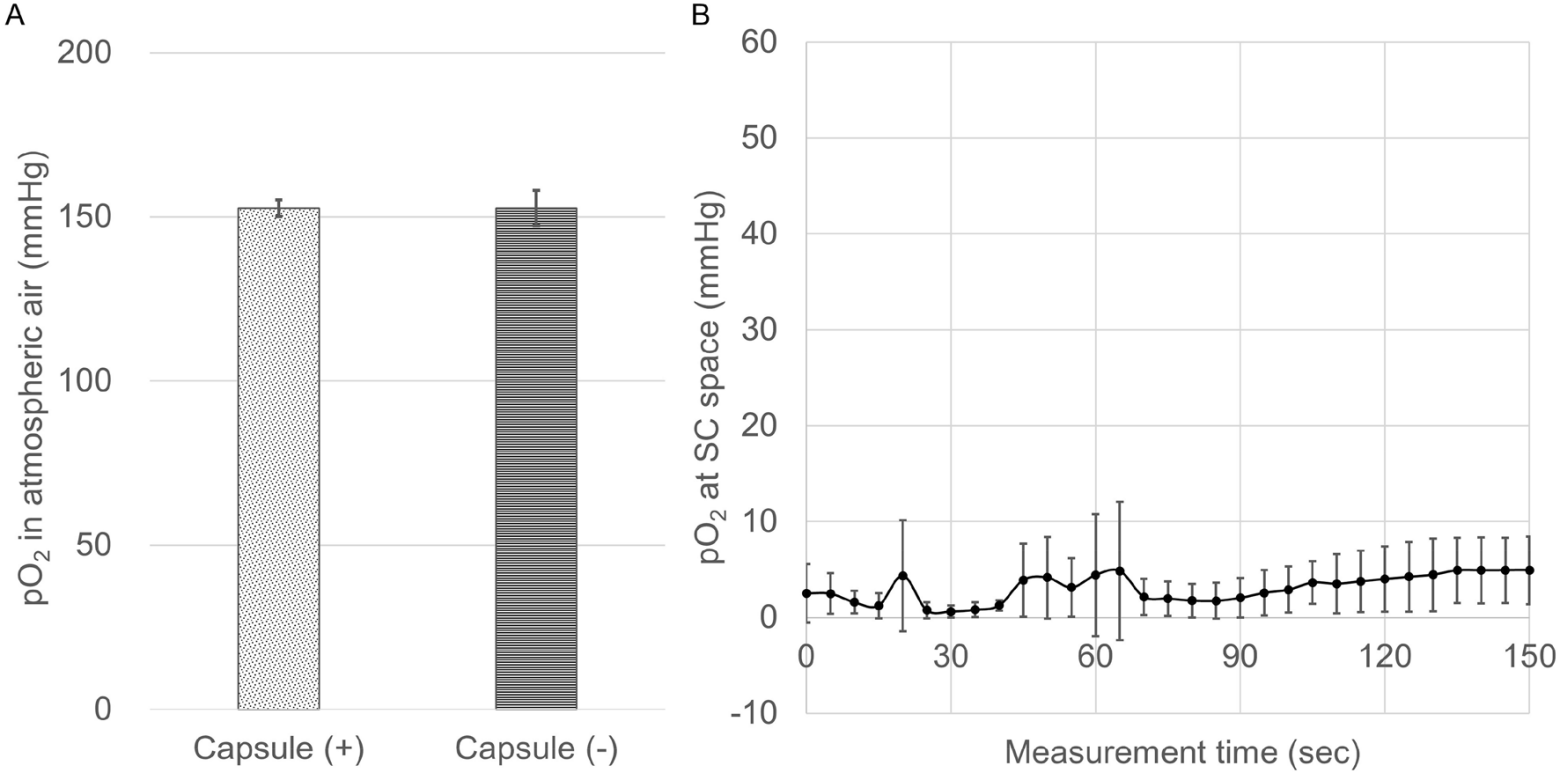
Influence of the interjacent subcutaneous capsule on the optical sensor system. (**A**) To examine the influence of the interjacent subcutaneous capsule on the optical sensor system, the pO_2_ of excised subcutaneous tissues at atmospheric air was measured by an optical sensor with or without capsules. There was no significant difference in the pO_2_ levels between the capsule (+) group (dotted bar; 152.6 ± 2.6 mmHg, n = 7) and capsule (−) group (horizontal stripe bar; 152.7 ± 5.4 mmHg, n = 7). p = 0.98 (Paired-samples *t* test). (**B**) Continuous measurement of pO_2_ at the SC 1 week after optical sensor implantation using the non-contact method. No increase of pO_2_ was detected during the 150-s observation period.

Since this optical measurement system was performed through the subcutaneous capsule, the influence of the interjacent subcutaneous capsule on the optical sensor was examined. First, the subcutaneous capsule was speculated to affect pO_2_ values, since the light transmittance of the capsule was not 100%. Therefore, the pO_2_ of excised subcutaneous tissues in atmospheric air was measured by an optical sensor with or without capsules. The pO_2_ levels of the two groups did not differ to a statistically significant extent (Fig. 2A, 152.6 ± 2.6 mmHg (n = 7) vs. 152.7 ± 5.4 mmHg (n = 7); p = 0.98). Second, because the thin capsule might permeate oxygen from the atmosphere and change the internal environment, we continuously measured the pO_2_ of the SC 1 week after optical sensor implantation using the non-contact method. No increase in the pO_2_ values was detected with time dependency during the 150-s observation period (Fig. 2B).

### Continuous measurement of pO_2_ at the subcutaneous space using the optical sensor system under several conditions of oxygenation

**Figure 3:**
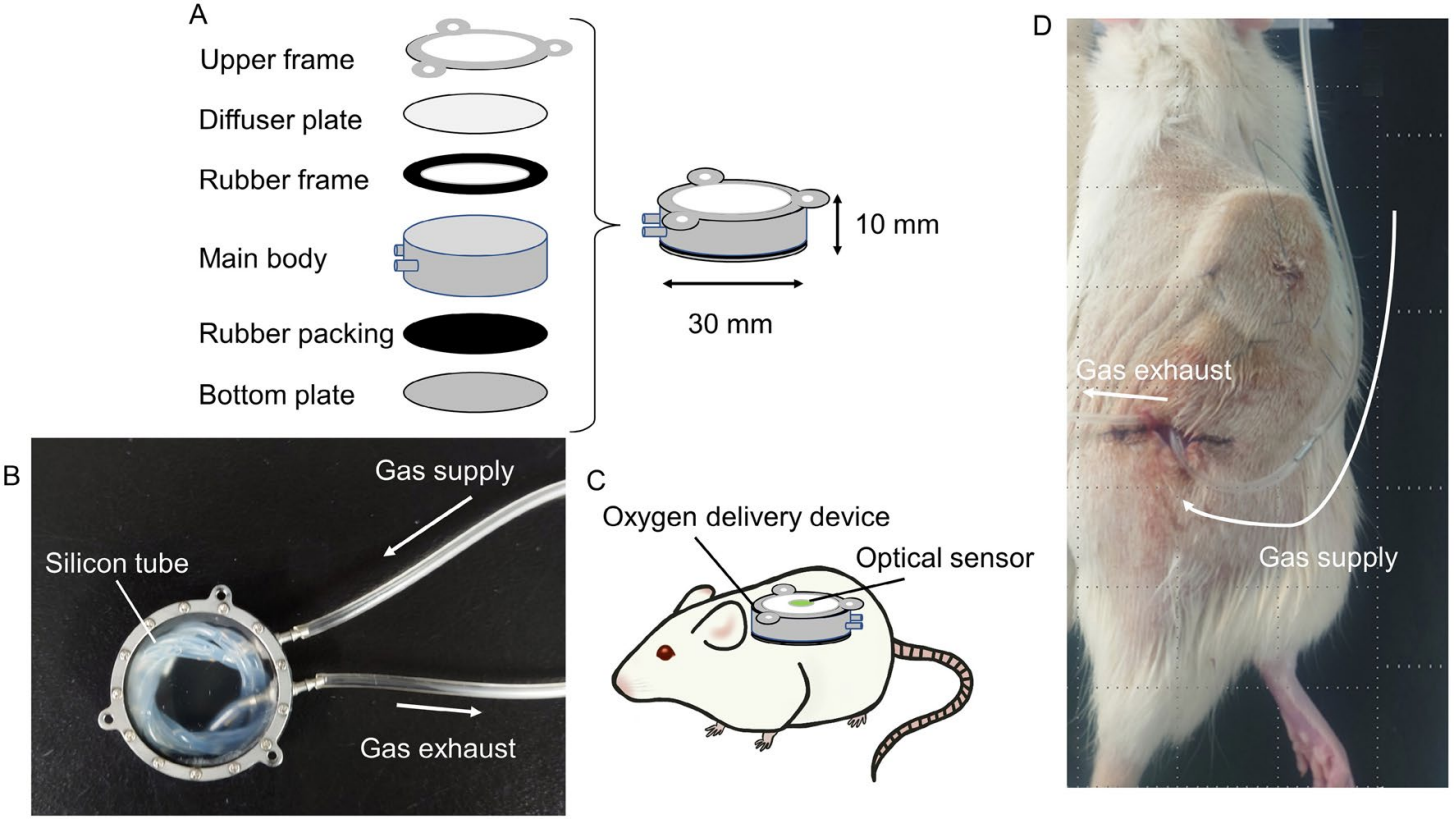
The oxygen delivery device and oxygenation procedure. (**A**) Overview of the components of the oxygen delivery device. The main body was filled with F6H8S5, and the oxygen contained in the F6H8S5 gradually diffused to the surrounding space. (**B**) Photograph of the oxygen delivery device. A silicon tube was connected to the cannulas inside the main body, and pure oxygen or ambient air was supplied to the device by a connecting tube outside the cannula (arrows) to oxygenate the F6H8S5. (**C**) Image of post-implantation of the oxygen delivery device for continuous measurement of pO_2_ at the subcutaneous space. An optical sensor was attached to the upper diffuser plate with silicone adhesive. (**D**) Oxygenation of the SC in rats. The oxygen delivery device was oxygenated after implantation into the SC. The gas was supplied through the tube (arrows) to oxygenate the implanted device.

**Figure 4:**
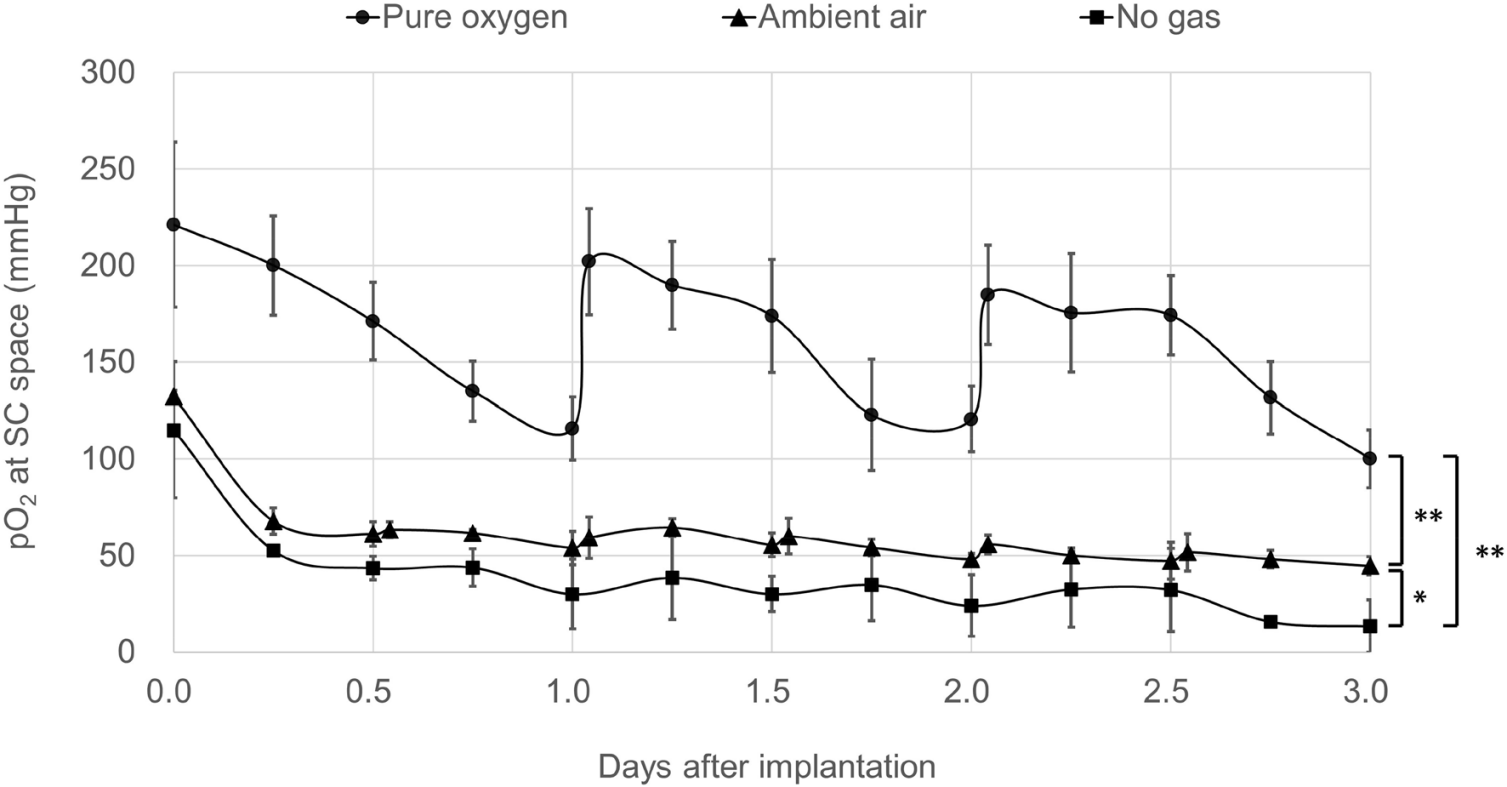
Continuous measurement of pO_2_ at the subcutaneous space using an optical sensor system under several oxygenation conditions. In the Pure oxygen group, the pO_2_ of the SC gradually decreased after device implantation, but promptly increased again after additional oxygenation. In the Pure oxygen group (circle, n = 6), the pO_2_ of the SC was maintained at significantly higher levels in comparison to the Ambient air (triangle, n = 5) and No gas (square, n = 6) groups during the observation period (**p < 0.01, One-way ANOVA with post-hoc Tukey–Kramer Test). The pO_2_ of the Ambient air group also remained significantly higher in comparison to the No gas group (*p < 0.05, One-way ANOVA with post-hoc Tukey–Kramer Test).

We developed the oxygen delivery device (Fig. 3A,B), and implanted it into rats subcutaneously. In the Pure oxygen group, the pO_2_ of the SC gradually decreased after device implantation, but promptly increased again after additional oxygenation (Fig. 4). In the Pure oxygen group (n = 6), the pO_2_ of the SC remained significantly higher in comparison to the Ambient air (n = 5) and No gas (n = 6) groups during the observation period (p < 0.01) (Fig. 4). The pO_2_ of the Ambient air group also remained significantly higher in comparison to the No gas group (p < 0.05) (Fig. 4).

### Verification of the utility of the optical sensor system by detecting the difference of the islet graft function between the Pure oxygen and No gas groups

**Figure 5:**
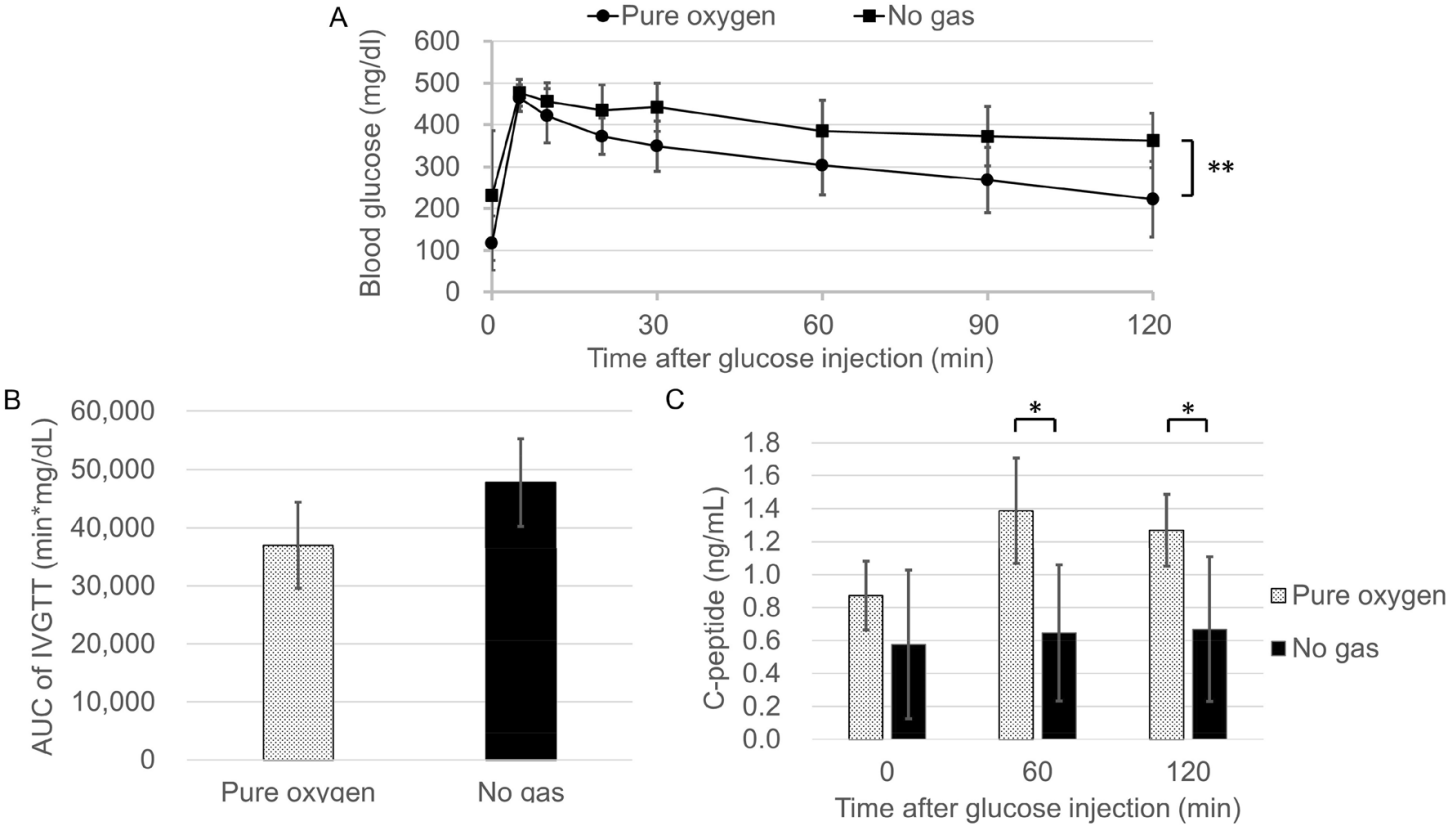
Difference of the islet graft function between the Pure oxygen and No gas groups. Profile of glucose tolerance in the Pure oxygen and No gas groups (n = 5, respectively). (**A**) The blood glucose changes of the intravenous glucose tolerance test (IVGTT). **p < 0.01 (Two-way ANOVA). (**B**) The area under the curve (AUC) of the IVGTT. p = 0.05 (Paired-samples *t* test). (**C**) The islet graft function examined by serum C-peptide levels during the IVGTT. The serum C-peptide levels in the Pure oxygen group (dotted bar) significantly increased at 60 and 120 min after glucose injection in comparison to the No gas group (black bar) (1.39 ± 0.32 ng/mL vs. 0.65 ± 0.41 ng/mL; 1.27 ± 0.22 ng/mL vs. 0.67 ± 0.44 ng/mL). *p < 0.05 (Mann–Whitney test).

**Figure 6:**
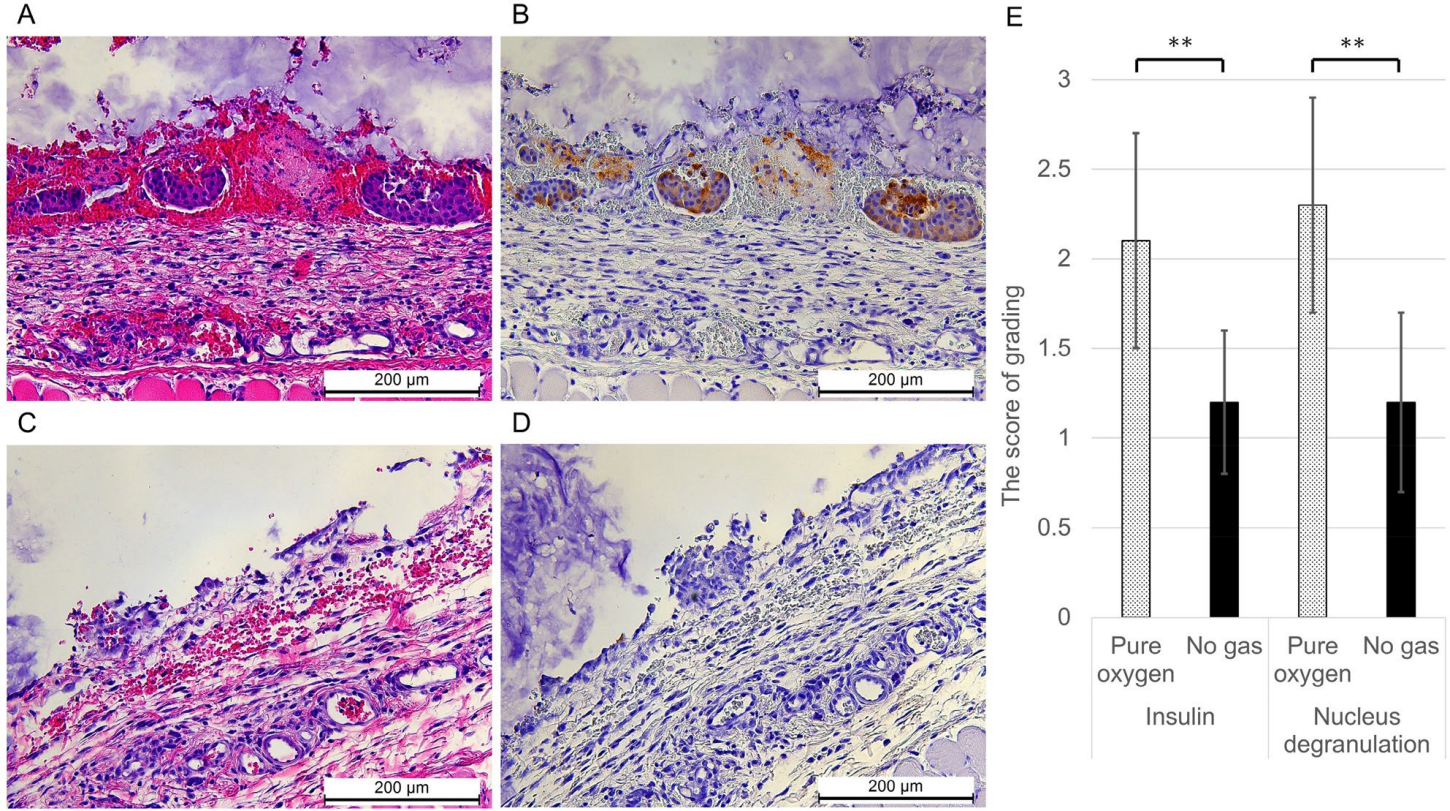
Immunohistochemical staining of transplanted islet grafts in the Pure oxygen and No gas groups. (**A**–**D**) Representative image of the graft islets of hematoxylin–eosin and insulin staining in the Pure oxygen group (**A**,**B**) and in the No gas group (**C**,**D**). Magnification: × 200, Scale bar: 200 μm. (**E**) Comparison of the scores of grading by the amount of insulin and the degree of nucleus degranulated. Both scores were significantly higher in the Pure oxygen group compared to the No gas group (2.1 ± 0.6 (n = 41) vs. 1.2 ± 0.4 (n = 24) in insulin grading, p < 0.01; 2.3 ± 0.6 (n = 41) vs. 1.2 ± 0.5 (n = 24) in nucleus grading, p < 0.01). **p < 0.01 (Student's *t* test).

The glucose tolerance of the transplanted recipients in the Pure oxygen group was significantly better in comparison to the No gas group (Fig. 5A, n = 5, respectively; p < 0.01), suggesting that superior functional engraftment of the islet grafts was seen in the Pure oxygen group. Likewise, the serum C-peptide levels in the Pure oxygen group significantly increased at 60 and 120 min after glucose injection in comparison to the No gas group (Fig. 5C, 60 min; 1.39 ± 0.32 ng/mL vs. 0.65 ± 0.41 ng/mL, n = 5, respectively, p < 0.05: 120 min; 1.27 ± 0.22 ng/mL vs. 0.67 ± 0.44 ng/mL, n = 5, respectively, p < 0.05). Immunohistochemistry also revealed that the islet grafts in the Pure oxygen group contained more viable beta cells than those in the No gas group (Fig. 6A,B). Quantitative assessment revealed that the scores of grading by the amount of insulin and the degree of nucleus degranulated were higher in the Pure oxygen group compared to the No gas group (2.1 ± 0.6 (n = 41) vs. 1.2 ± 0.4 (n = 24) in insulin grading, p < 0.01; 2.3 ± 0.6 (n = 41) vs. 1.2 ± 0.5 (n = 24) in nucleus grading, p < 0.01).

### The difference of islet graft viability between the Ambient and No gas groups

**Figure 7:**
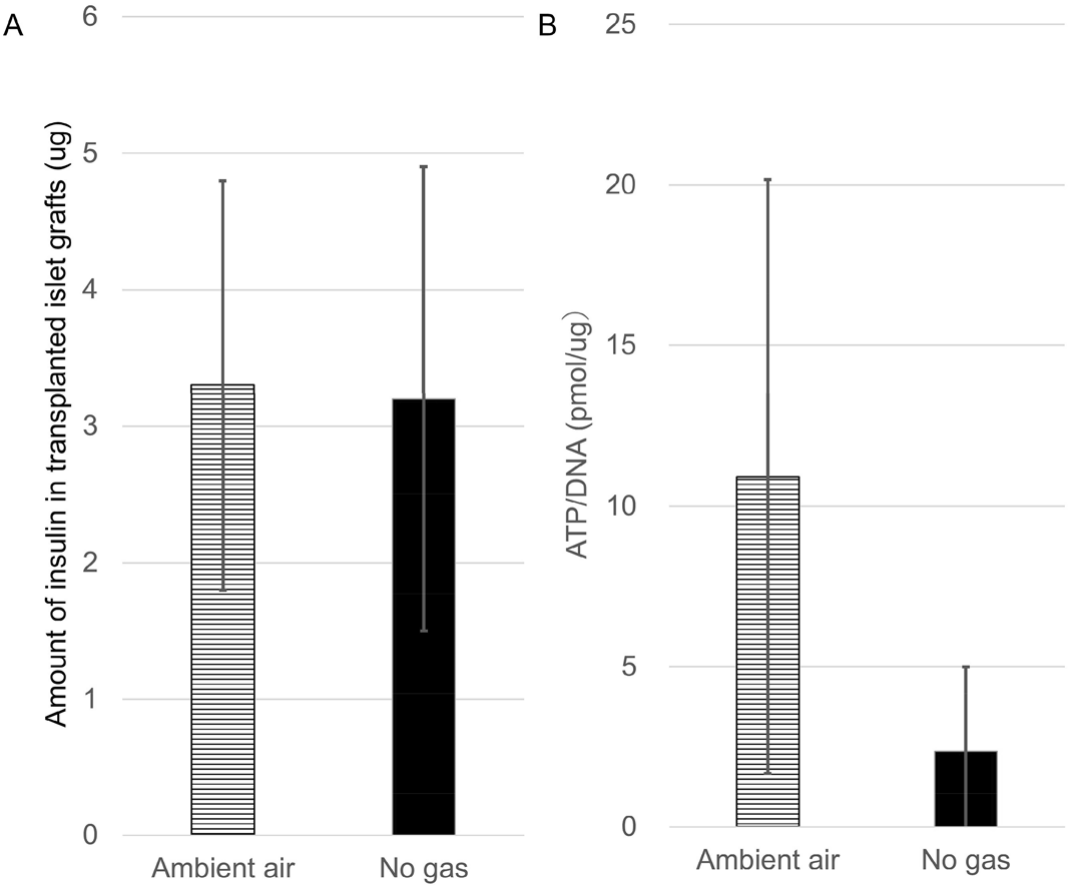
Difference of islet graft viability between the Ambient and No gas groups. (**A**) The amount of insulin in the transplanted islet grafts. Three days after transplantation at the SC, the islet grafts were retrieved, and the amount of insulin was evaluated in the Ambient air (horizontal stripe bar; 3.3 ± 1.5 μg, n = 9) and No gas (black bar; 3.2 ± 1.7 μg, n = 9) groups. p = 0.81 (Paired-samples *t* test). (**B**) The ATP/DNA of the transplanted islet grafts in the Ambient air (horizontal stripe bar; 10.9 ± 9.3 pmol/μg, n = 5) and No gas (black bar; 2.4 ± 2.6 pmol/μg, n = 5) groups. p = 0.07 (Paired-samples *t* test).

In terms of the amount of insulin in the islet grafts, there was no significant difference between the Ambient air and No gas groups (Fig. 7A, 3.3 ± 1.5 μg vs. 3.2 ± 1.7 μg, n = 9, respectively; p = 0.81). However, the ATP/DNA of islet grafts in the Ambient air group tended to be higher than that in the No gas group (Fig. 7B, 10.9 ± 9.3 pmol/μg vs. 2.4 ± 2.6 pmol/μg, n = 5, respectively; p = 0.07), although the difference did not reach statistical significance. In these assays, collagen gel was used to retrieve islet grafts from the recipients, and treated by collagenase and thermolysin to pick up islet grafts from them. Therefore, islets were cultured in collagenase and thermolysin solution (Enzyme group) or Hank’s balanced salt solution (HBSS group), then the ATP/DNA assay was performed to evaluate the influence of enzymes on the islet grafts. No difference was observed between the two groups (see Supplemental Fig. S1), suggesting that the enzymes used in these assays had no influence on the islet viability.

## Discussion

Our findings revealed that the pO_2_ at the SC, which was previously reported to be equivalent to other transplant sites, was extremely low in comparison to previous reports^28,30^. These results are consistent with the outcomes reported by Einstein^32^, who measured the pO_2_ of encapsulated tissue-engineered grafts implanted into a dorsal subcutaneous pocket of a rat in vivo by magnetic resonance relaxometry. Of particular interest, unlike a conventional Clark-type needle probe, both systems are non-invasive and involve no contact with target tissues. Polarographic techniques using a Clark-type needle probe remain the gold standard for in vivo oxygen measurements; however, these needle-type sensing methods are invasive and may introduce oxygen to the system during the mechanically invasive measurement procedure. More importantly, these methods have the potential to overestimate the pO_2_ values of target tissues, most likely due to an accidental puncture of the indwelling micro-vessels in the target tissues. In contrast, optical sensors can measure tissue oxygen less invasively, and theoretically make it possible to apply without contact against the target tissues. These features make them an attractive alternative to needle-type oximetry. To our knowledge, this is the first report to demonstrate that subcutaneous pO_2_ measured less invasively by optical sensors is lower than that measured by the current gold standard method. Considering that measuring the pO_2_ at the SC is important for subcutaneous islet transplantation to resolve the deleterious damage to the islet grafts due to hypoxia, the present novel technique can make up for the drawbacks of previous methods, and may therefore be regarded as more practical.

Subcutaneous transplantation is undoubtedly an ideal and reasonable site for islet transplantation due to several advantages, including its minimal invasiveness, replicability, and easy monitoring and removability of grafts in the event that complications of stem cell-derived cellular therapy occur in the post-transplantation period. However, poor engraftment, mainly due to low vascularity and hypoxia—in comparison to the conventional intrahepatic site—hinders its prevalence. Several methods have been introduced to overcome these problems. Biocompatible materials are one such candidate^33^. Our group has so far focused on biomaterials to induce efficient prevascularization in a mouse model, and reported that a recombinant peptide was effective for improving islet engraftment in subcutaneous transplantation, despite the fact that newly-constructed vessels surrounding the islet grafts were not as enhanced as we had expected^34^. This indicates the possibility that the impact of hypoxia may be more crucial for islet grafts transplanted into the subcutaneous site than anticipated^35^. Islet survival depends on oxygen diffusion through the surrounding tissue, as well as oxygen transported by hemoglobin^25^. Since a lack of oxygen is the most critical issue for preventing islet engraftment initially after transplantation, the delivery of exogenous oxygen may be effective for supporting the survival and function of islet grafts until revascularization occurs. In fact, unique in vivo treatment methods have been developed and tested in animal models, including localized oxygenation using a biocompatible device and bioartificial pancreas^27,29^ and systemic oxygen inhalation^28^. To some extent, the improvement of islet engraftment in subcutaneous transplantation has been realized by these methods. The data in our study (Fig. 5) are in accord with these previous reports, and demonstrate that the optical sensor system was effective for measuring tissue oxygen pressure in vivo.

The RS is also a promising site for islet transplantation in animal models, and this transplantation site is well recognized as an experimental procedure by most investigators in this field^18,24,36^. In our experiments, the pO_2_ of the RS were equivalent to those of the portal vein in previous reports^30,37^, but were obviously higher in comparison to the SC (Fig. 1C). Considering that the islet engraftment of the RS is much better than that of the SC, our experimental result makes sense. Of particular note, Bochenek et al. previously reported that “Interestingly, the subcutaneous site yielded higher pO_2_ levels than anticipated (39.0 ± 2.1 mmHg)”^30^. Their estimated pO_2_ values of the SC were almost equivalent to those measured using needle probe type sensor in the present study. Furthermore, they also demonstrated that pO_2_ values of the SC are higher than those of intramuscular space or intraperitoneal space, and equivalent to liver tissues or pancreatic tissues. It is quite difficult to logically explain these unexpected outcomes. Taken together, we speculate that these discrepancies may be based on an accidental puncture of the indwelling micro-vessels in the target tissues using needle-type sensors.

In this study, we confirmed the utility of the optical sensor system by correlating the pO_2_ values with the transplanted islet function using an implantable oxygen delivery device (Fig. 5). Interestingly, this novel technique revealed that the energy charge, but not the amount of insulin, of subcutaneously transplanted islets in the Ambient air group tended to be higher in comparison to the No gas group (Fig. 7A,B), suggesting that the islet viability was more damaged by the hypoxic condition at the SC than we had anticipated. In other words, this finding indicates that islet engraftment may substantially improve if the pO_2_ levels reached those of the RS.

The present study was associated with some limitations. First, in an in vitro islet viability assay, the use of collagen gel as a scaffold for islets was needed to retrieve islet grafts from the recipients. Although this was effective, some islet grafts may be lost during the recovery process. Furthermore, collagenase and thermolysin, which were used for islet collection during recovery from collagen gel, may affect the viability of retrieved islets. To examine this concern, healthy islets were cultured in HBSS solution with or without collagenase and thermolysin, and evaluated by an ATP/DNA assay (Fig. 7C). The result showed no significant difference between the two groups; thus, in our opinion, this method is valid for the assessment of graft viability. However, in vivo imaging of islet grafts is a topic of interest for our next study. Second, the significance of confounding factors other than oxygenation, such as extracellular matrix^34^, inflammation^38,39^, and the condition of blood vessels and local blood flow at the measurement site, could not be evaluated in this study. To improve the outcome of subcutaneous islet transplantation, in addition to optimizing oxygenation using the present novel technique, the amelioration of extracellular matrix and/or inflammation would be warranted as a next step. Third, the in vivo graft function was only evaluated between the Pure oxygen and No gas groups, but not in the Ambient air group. We also assessed the graft function only for short period. Future studies are needed to evaluate graft function under several conditions in terms of oxygen supply for longer post-implantation period. Fourth, in vivo investigations in the present study were insufficient to completely justify the present new method. We have just performed these in vivo studies to make sure that pO_2_ values measured using our new method could parallelly work according to the transplant outcomes. However, by introducing our new method, these investigations revealed that pO_2_ values of subcutaneous tissue and renal subcapsular space, which have been reported equivalent by using conventional methods, were apparently distinct and this difference might at least in part impact on the viability of islet grafts. In addition, the pO_2_ values at the subcutaneous site measured by using other type of non-contact method, reported by Einstein et al.^32^, are consistent with our measurement values. Furthermore, it is logically unreasonable to think that pO_2_ values at the transplant site are not associated with the causes of clear difference in the outcomes between subcutaneous transplantation and renal subcapsular transplantation. Taken together, we believe that the pO_2_ values in the present study are logical and reliable, though they can’t be a complete justification. Further investigations on pO_2_ values at the subcutaneous tissue using several other types of non-contact systems may be useful to justify our new method.

In conclusion, we developed a novel method for measuring tissue oxygen pressure less invasively in comparison to conventional methods, and found that the pO_2_ of the SC was extremely lower in comparison to other transplant sites. We propose that the increase in pO_2_ of the SC using the present technique in combination with optimization of the transplant site may contribute to improving the efficiency of subcutaneous islet transplantation.

## Materials and methods

### Animals

All animals in the present study were handled in accordance with the Animal Research: Reporting of In Vivo Experiments (ARRIVE) guidelines, the Guide for the Care and Use of Laboratory Animals published by the National Institutes of Health^40^. All experimental protocols of the present study (protocol ID: 2019 MdA-312) were approved by the animal experimental committee in the Tohoku University. Male Lewis rats (age: 8–14 weeks, body weight: 283.7 ± 26.3 g; Japan SLC Inc., Shizuoka, Japan) were used for pO_2_ measurement, donors and recipients. All rats were housed under specific pathogen-free conditions and had free access to food and water. All surgeries were performed under general anesthesia using isoflurane, and all efforts were made to minimize suffering.

### Measurement of oxygen partial pressure at the subcutaneous space and renal subcapsular space

To measure native pO_2_, we adopted optical oxygen sensors, called Redflash Technology (FireSting O_2_; PyroScience, Aachen, Germany). A contactless oxygen sensor (PyroScience, Aachen, Germany) was implanted into the SC (Fig. 1A) and RS (Fig. 1B) of rats under general anesthesia. In briefly, this system comprised of two important parts: an oxygen sensitive sensor and a read-out device (oxygen meter). The oxygen sensitive sensor emitted oxygen dependent luminescence when it was irradiated excitation light. The oxygen meter detected the emitted light of the sensor which was dependent on the oxygen partial pressure at each site. The sensor was calibrated by 2-point calibration using anoxic water prepared by adding SO_3_ for 0 mmHg in factory and ambient air for 157 mmHg in laboratory at room temperature before each experiment. After waiting almost 1 week until the improvement of inflammation, the pO_2_ of each site was measured by a non-puncture-type optical fiber through the subcutaneous capsules (Fig. 1C). Likewise, pO_2_ was measured using a puncture-type optical fiber (Fig. 1D). In both methods, measurements were taken every 5 s for 1 min, and the average value was regarded as the pO_2_ value and compared.

### Verification of the influence of the interjacent subcutaneous capsule on the optical sensor system

To evaluate the effect of translucency, the subcutaneous capsule was removed from the rats and the pO_2_ of the atmosphere was measured through the capsule. As a control, an optical sensor was also measured without the subcutaneous capsule (Fig. 2A). To evaluate the effect of oxygen permeability of the thin capsule, the subcutaneous pO_2_ was continuously measured for 150 s with the capsule exposed to the atmosphere, and we examined whether or not the measured values increased with time (Fig. 2B).

### Design and concept of oxygen delivery device

The oxygen delivery device was composed of a stainless steel main body, bottom plate and two cannulas, and oxygen releasable upper diffuser plate made from polymethylpentene (PMP) (Umihira, Kyoto, Japan) (Fig. 3A). A 20-cm silicon tube (Silicone Tube 1 × 1.5, outer diameter = 1.5 mm, inner diameter = 1.0 mm, AS ONE, Osaka, Japan) was connected to the cannulas inside the main body, and perfluorohexyloctane (F6H8S5; Novaliq GmbH, Heidelberg, Germany) were filled into the device (Fig. 3B). For F6H8S5 oxygenation, pure oxygen or ambient air was supplied to the device by a connecting tube outside the cannula, and the contained oxygen was slowly released through the diffuser plate to the surroundings. To prevent leakage of F6H8S5, rubber packing was set at the PMP and bottom plates.

### Continuous measurement of pO_2_ at subcutaneous space and experimental groups

To measure the oxygen concentration at the same site where the islets were transplanted at SC, an optical sensor was attached to the upper diffuser plate of the oxygen delivery device with silicone adhesive (Fig. 3C). The oxygen delivery device was implanted subcutaneously into the upper back of the anesthetized rats and fixed with a thread. In addition, the gas supply and exhaust tube tips were connected and the connecting portion was placed subcutaneously in the lower part of the back. The skin was incised under anesthesia and the connecting portion was taken out when gas was supplied (Fig. 3D). After the gas supply was completed, incisions were closed and returned to the subcutaneous pocket. Three groups were made in this experiment (Fig. 4). In the Pure oxygen group, which aimed to improve the hypoxic environment under the skin, the oxygen delivery device was oxygenated with 0.1 L/min pure oxygen for 2 h, then implanted subcutaneously into rats and re-oxygenated with 0.1 L/min pure oxygen for 1 h every 24 h after implantation. In the Ambient air group, which aimed to obtain equivalent pO_2_ values to the renal subcapsular space, the oxygen delivery device was implanted subcutaneously without preoxygenation and moderately oxygenated by 0.1 L/min atmospheric air for 1 h every 12 h. In the No gas group, which was served as a negative control, the bottom of the diffuser plate was covered by a rubber plate to prevent oxygenation of the surroundings, and the device was not oxygenated before or after implantation. Then—in order to match the conditions of the other groups—anesthesia was performed for the same time as the gas supply group. The pO_2_ of the SC in each group after device implantation was measured. An oxygen delivery device with a contactless oxygen sensor attached to the upper diffuser plate with silicone adhesive was embedded in the rat. Then, every 6 h, the skin directly above the sensor was incised under anesthesia so as not to break the subcutaneous capsule, and the pO_2_ of the SC was measured by a non-contact method for 3 days. The measurement incision was ligated with a thread after the completion of measurement. The Pure oxygen group and Ambient air group were measured at 1 h after oxygenation.

### The induction and diagnosis of diabetes in the recipients

Diabetes was induced by the intravenous injection of 70 mg/kg streptozotocin (STZ; SIGMA-ALDRICH, Inc, MO, USA) according to the previous report^41^. Rats whose non-fasting blood glucose levels were ≥ 400 mg/dL on two consecutive measurements were considered diabetic.

### Islet isolation

Islet isolation and culturing were performed as previously described^42^. In brief, the bile duct was identified and clamped at the papilla of Vater. Ten milliliters of cold HBSS containing 0.8 mg/mL collagenase type V (Sigma Chemicals, St. Louis, MO, USA) was injected into the common bile duct leading to the pancreas. The pancreas was removed and incubated in a water bath at 37 °C for 12 min before being digested, and the cell suspension was washed 3 times in cold HBSS and centrifuged for 1 min. Density-gradient centrifugation was performed for 10 min using a Histopaque-1119 (Sigma Diagnostics, St. Louis, MO, USA) and Lymphoprep™ (Axis-Shiled, Oslo, Norway) to isolate pancreatic islets. The islets were cultured in Roswell Park Memorial Institute-1640 (RPMI) medium containing 5.5 mmol/L glucose and 10% fetal bovine serum at 37 °C in 5% CO_2_ and humidified air overnight before transplantation.

### Subcutaneous islet transplantation and intravenous glucose tolerance test

After implanting the oxygen delivery device into the subcutaneous space of diabetic recipient rats, 6000 islet equivalents (IEQs) of syngeneic rat islets were transplanted into subcutaneous site uniformly distributed just above the 30-mm-diameter diffuser plate of the oxygen delivery device using a plastic cannula-type 18G puncture needle and a gastight syringe (Hamilton Co., Reno, NV, USA)^34^ to receive oxygen supply directly from the device. Then, the transplanted islets were covered with a seprafilm (KAKEN PHARMACEUTICAL CO., LTD., Tokyo, Japan) to prevent them from moving away and to fix them to the transplant site. An intravenous glucose tolerance test (IVGTT) and serum C-peptide (a byproduct of insulin production) measurement were performed as in vivo functional assessments of transplanted islets. The IVGTT was performed as previously described^43^. D-glucose (3.0 g/kg) was intravenously infused, and the blood glucose concentrations were determined before and at 5, 10, 20, 30, 60, 90, and 120 min after the injection of glucose. Rat serum C-peptide was measured before and at 60 and 120 min after glucose injection using a rat C-peptide ELISA kit (Mercodia, Uppsala, Sweden). These assessments were performed 3 days after transplantation in the Pure oxygen and No gas groups (n = 5, respectively).

### Immunohistochemical analyses

At the time of removal of oxygen delivery device, the recipient subcutaneous tissues around transplanted area were retrieved, fixed with 4% paraformaldehyde, and embedded in paraffin for immunohistochemical staining. Insulin staining was performed using anti-insulin antibodies (Dako, Glostrup, Denmark) as previously described^44^. The degree of the nuclear degranulation and insulin staining in grafts were scored in three grades at 200-fold magnification, and quantitative assessments were made by comparing the grade in each islet between the Pure oxygen and the No gas groups. In insulin staining, Grades 1, 2, and 3 meant that the islets were unstained, partially, or diffuse stained, respectively. In hematoxylin–eosin staining, Grades 1, 2, and 3 meant that the islets were broken and most of the nuclei were degranulated, that partially degranulated but less than 70% or more than 70% of the nuclei remain, respectively. More than 20 sections from each experimental group were evaluated.

### Insulin extraction from the transplanted islet grafts

Insulin extraction from the transplanted islet grafts was performed to compare the residual amount of the grafts, as previously described^45^. In brief, collagen gel was prepared using 0.5% Native Collagen Acid Solution (AteloCell IA-C50; Koken Co., Ltd., Tokyo, Japan) according to the commercial instruction. Then, 0.75 mL of collagen gel and 1200 IEQs of syngeneic rat islets were mixed, placed on the upper diffuser plate of an oxygen delivery device, heated to 37 °C for 30 min to cure the gel, and integrated with the device. The integrated device was then embedded in the back of the recipient rat. Three days after transplantation, the collagen gel samples were collected and homogenized with 1 mL of deionized water at 4 °C, with 24.6 × 1000 rpm for 2 min. After adding 4 mL of deionized water and 12.5 mL of 0.18 M HCl in 96% ethanol, the homogenate was stored at 4 °C for 24 h and was then centrifuged at 1300 × *g* for 5 min. The resulting supernatant was stored at − 80 °C. The insulin concentration in the supernatant was evaluated using a Rat Insulin ELISA kit (Mercodia AB, Uppsala, Sweden), as previously described^46^.

### The ATP/DNA assay

The ATP/DNA assay was performed to compare the energy status of the transplant islet grafts. The procedure for preparing, transplanting, and removing the islet grafts was the same as that for insulin extraction. The collagen gel sample was immersed in a 2 mL HBSS solution containing 15 mg of collagenase and 0.4 mg of thermolysin (Liberase MTF C/T; Roche Diagnostics, Roche Applied Science, Indianapolis, IN, USA) at 37 °C for 30 min to dissolve and the islets were retrieved. More than 40 islets were picked up and cultured in RPMI medium containing 5.5 mmol/L glucose and 10% fetal bovine serum at 37 °C in 5% CO_2_ and humidified air over one hour. The ATP/DNA assay for the islet grafts was performed as previously described^47,48^.

### Statistical analyses

All data are expressed as the mean ± standard deviation. All statistical analyses were performed using the JMP pro 16 software program (SAS Institute Inc., Cary, NC, USA). Statistical significance was determined using Student’s *t* test, a paired-samples *t* test, Mann–Whitney test, a one-way analysis of variance (ANOVA) followed by Tukey–Kramer post hoc test, and a two-way ANOVA. p values of < 0.05 were considered to indicate statistical significance.

## Supplementary Information

Supplementary Figure S1.

## Data Availability

All data generated or analyzed in the present study were included in this published manuscript.

## Abbreviations

ANOVA: Analysis of variance
AUC: Area under the curve
F6H8S5: Perfluorohexyloctane
HBSS: Hank’s balanced salt solution
IEQs: Islet equivalents
IVGTT: Intravenous glucose tolerance test
PMP: Polymethylpentene
pO_2_: Oxygen partial pressure
RPMI: Roswell Park Memorial Institute-1640
RS: Renal subcapsular space
SC: Subcutaneous space
STZ: Streptozotocin
T1D: Type 1 diabetes
