# Supplementary material for: Development of a novel method for measuring tissue oxygen pressure to improve the hypoxic condition in subcutaneous islet transplantation
Source: Sci Rep. 2022 Aug 30;12:14731. doi: 10.1038/s41598-022-19189-2 (PMC9427780; doi:10.1038/s41598-022-19189-2)
Supplement: Supplementary file 1 — Supplementary Figure S1. [file 41598_2022_19189_MOESM1_ESM.pdf]

Supplemental Information

Development of a novel method for measuring tissue oxygen pressure to improve the hypoxic condition in subcutaneous islet transplantation

Hiroaki Mitsugashira, MD[1], Takehiro Imura[2], Akiko Inagaki, PhD[2], Yukiko Endo, MD[1], Takumi Katano, MD[2], Ryusuke Saito, MD[1], Shigehito Miyagi, MD, PhD[1], Kimiko Watanabe, PhD[2], Takashi Kamei, MD, PhD[1], Michiaki Unno, MD, PhD[1], *Masafumi Goto, MD, PhD[1,2]

[1]Department of Surgery, Tohoku University Graduate School of Medicine, 980-0872, Sendai, Japan

[2]Division of Transplantation and Regenerative Medicine, Tohoku University Graduate School of Medicine, 980-8575, Sendai, Japan

*Correspondence to masafumi.goto.c6@tohoku.ac.jp

Supplemental Figure Legends.

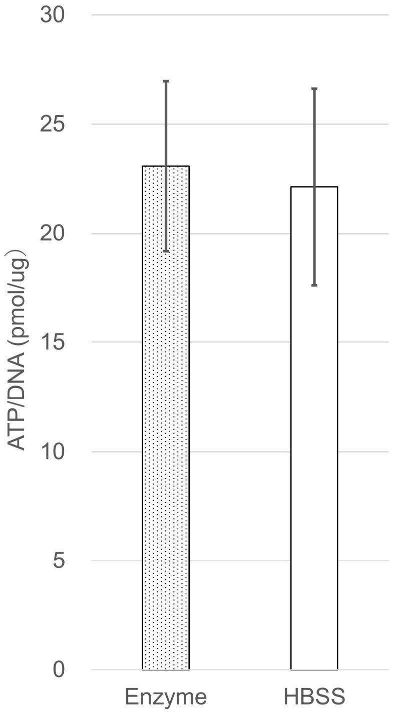

Supplemental Figure S1. Evaluation of the influence of collagenase and/or thermolysin on the islet grafts. The ATP/DNA assay revealed no difference between the Enzyme (dotted bar; 23.1±3.9 pmol/μg, n=6) and HBSS (white bar; 22.1±4.5 pmol/μg, n=6) groups. p=0.64 (Paired-samples t-test).
